# Delayed Onset Muscle Soreness and Critical Neural Microdamage-Derived Neuroinflammation

**DOI:** 10.3390/biom12091207

**Published:** 2022-08-31

**Authors:** Balázs Sonkodi

**Affiliations:** Department of Health Sciences and Sport Medicine, Hungarian University of Sports Science, 1123 Budapest, Hungary; bsonkodi@gmail.com

**Keywords:** delayed onset muscle soreness, Piezo2 ion channel, proprioception, neuroinflammation, nuclear factor-kappa B, noncontact injury

## Abstract

Piezo2 transmembrane excitatory mechanosensitive ion channels were identified as the principal mechanotransduction channels for proprioception. Recently, it was postulated that Piezo2 channels could be acutely microdamaged on an autologous basis at proprioceptive Type Ia terminals in a cognitive demand-induced acute stress response time window when unaccustomed or strenuous eccentric contractions are executed. One consequence of this proposed transient Piezo2 microinjury could be a VGLUT1/Ia synaptic disconnection on motoneurons, as we can learn from platinum-analogue chemotherapy. A secondary, harsher injury phase with the involvement of polymodal Aδ and nociceptive C-fibers could follow the primary impairment of proprioception of delayed onset muscle soreness. Repetitive reinjury of these channels in the form of repeated bout effects is proposed to be the tertiary injury phase. Notably, the use of proprioception is associated with motor learning and memory. The impairment of the monosynaptic static phase firing sensory encoding of the affected stretch reflex could be the immediate consequence of the proposed Piezo2 microdamage leading to impaired proprioception, exaggerated contractions and reduced range of motion. These transient Piezo2 channelopathies in the primary afferent terminals could constitute the critical gateway to the pathophysiology of delayed onset muscle soreness. Correspondingly, fatiguing eccentric contraction-based pathological hyperexcitation of the Type Ia afferents induces reactive oxygen species production-associated neuroinflammation and neuronal activation in the spinal cord of delayed onset muscle soreness.

## 1. Introduction

Delayed onset muscle soreness (DOMS) has been defined as delayed onset soreness, muscle stiffness, swelling, loss of force-generating capacity, reduced joint range of motion, and decreased proprioceptive function [1]. Unaccustomed or strenuous exercises involving eccentric contractions are known to induce DOMS with neuromuscular changes for several days [2,3,4]. The pain of DOMS is not felt for approximately 8 h, peaks 1 or 2 days later [3], and subsides within 7 days after exercise [5]. Theodore Hough first described DOMS in 1902, attributing soreness to ruptures in the muscles [6]. However, the mechanism of DOMS is still far from entirely understood. Several theories, such as lactic acid, muscle spasm, inflammation, connective tissue damage, muscle damage, enzyme efflux and most recently neuronal damage and the Piezo2 microdamage theory attempt to explain the mechanism of DOMS [7,8,9], but no single theory or factor has answered the question entirely. Notably, DOMS is different from pain experienced during or immediately after exercise [10], as DOMS could be induced without muscle damage [11], similarly to vibration [12]. Furthermore, exercise-induced muscle damage could exist without DOMS, and even earlier findings seemed to conclude that DOMS inducement is independent of inflammation [13] or that it is not essential for DOMS [5].

The current review highlights the critical pathways in the DOMS mechanism, especially focusing on proprioceptive sensory microdamage as a principal mechanism leading to neuroinflammation.

## 2. Neural Microdamage of DOMS

It is important to note certain milestones that led to the neuronal microdamage theory of DOMS. Weerakkody et al. was the first to demonstrate the contribution of muscle spindle-derived proprioceptors in DOMS [12,14]. Later, Torres et al. showed that reduced proprioception in DOMS is muscle spindle-induced; however, they theorized that the eccentric exercise-derived damage is from intrafusal muscle fibers [2]. Proske and Gandevia reported that eccentric exercise indeed damages proprioception [15].

A significant milestone was the novel concept, put forward by Bennet et al., of neuronal terminal lesions, called terminal arbor degeneration (TAD), that could be learned from paclitaxel chemotherapy [16]. Kouzaki et al. demonstrated that eccentric exercise-induced muscle damage increased the M-wave latency and implicated reversible motoneuronal damage, but excluded muscle spindle origin [17]. However, it is known from Vincent et al. that platinum analogue chemotherapy causes complex Type Ia proprioceptive impairment, and this lesion could evolve in an acute and chronic fashion as well [18,19]. Furthermore, Alvarez et al. and Bullinger et al. showed permanent central synaptic disconnection of proprioceptors from motoneurons after nerve injury, and this phenomenon is associated with the loss of vesicular glutamate transporter (VGLUT) 1/Ia synapses on motoneurons [20,21]. Sonkodi et al. suggested that the muscle spindle origin of increased M-wave latency on motoneurons after eccentric exercise-induced muscle damage should not be excluded [22] and even proposed that the transiently impaired muscle spindle-derived proprioceptors and the resultant synaptic disconnection on motoneurons are responsible for the increased M-wave latency in DOMS, in line with the findings of Vincent, Alvarez and Bullinger et al. [19,20,21,22].

The significant findings of Murase et al. that bradykinin and nerve growth factor (NGF) are pivotal in DOMS inducement were supplemented by Kubo et al. and Ota et al., who showed that C-fiber and transient receptor potential vanilloid (TRPV) 1/TRPV4 also play an essential role in the DOMS mechanism [23,24,25].

The earlier research work of Bewick et al. was fulfilled by Than et al. on glutamatergic autoexcitation in muscle spindles, substantially helping the understanding of glutamate-based signaling of static phase firing sensory encoding [26,27].

Sonkodi et al. hypothesized that DOMS is an acute stress response-induced acute compression proprioceptive axonopathy derived from muscle spindles [8]. Sonkodi et al. even suggested that the primary microinjury of the dichotomous noncontact injury mechanism of DOMS is a transient Piezo2 channelopathy at the peripheral terminal of Type Ia proprioceptors [9]. Notably, Piezo2 channels are shown to be the principal mechanotransduction channels for proprioception [28], involved in vibration sensing [29], and bone-derived osteocalcin was demonstrated to mediate an acute stress response [30].

Sonkodi also theorized that the result of acute Type Ia proprioceptive terminal microinjury is represented in the delayed latency of the medium latency response (MLR) of the affected stretch reflex due to a switch of monosynaptic Type Ia static phase firing sensory encoding to polysynaptic Type II static phase firing encoding [9,31,32]. Notably, MLR is commonly viewed by scientists as dominantly Type II afferent mediated [33,34,35,36,37,38,39,40,41]. Indeed, Sonkodi et al. demonstrated that delayed latency of MLR is a consequence of unaccustomed fatiguing exercise that induced DOMS [32].

Most recently, Borghi et al. showed that intense acute DOMS-inducing swimming causes spinal cord neuroinflammation [42], as was hypothesized by Sonkodi et al. [8,31].

## 3. Transient Piezo2 Channelopathy

In order to understand the proposed dichotomous noncontact injury mechanism of DOMS, first, the suggested Piezo2 gateway to pathophysiology should be introduced.

### 3.1. Piezo Ion Channels

Piezo proteins are giant force-gated excitatory mechanosensitive and nonselective ion channels with numerous transmembrane segments [43]. In fact, they are the largest pore-forming transmembrane ion channel proteins known so far [44]. They are responsible for mechanotransducing life-sustaining signals, such as touch sensation, proprioception and cardiovascular regulation [45]. Two types of Piezo proteins are found in humans, and both Piezo1 and Piezo2 channels have interesting structural properties, such as their propeller blades and fairly similar conformations [46]. Nevertheless, the exact topology and functions, such as pore formation, mechanical force detection and gating, are not entirely understood [43]. However, recent findings are rapidly emerging to provide answers to these questions.

Piezo1 channels have an important role in mechanotransduction to maintain homeostasis in peripheral tissues such as in cartilage [47], blood pressure regulation [48], urinary osmolarity [49], and dorsal root ganglion (DRG) neuron physiology [50]. Moreover, Piezo1 channels take part in cell alignment based on their shear stress sensor capability [51,52], and this signaling could have relevance in the loading of tissues, such as muscles, bones and joints, not to mention their remodeling [53]. In contrast, Piezo2 ion channels contribute to maintaining homeostasis in somatosensory neurons [51,54,55].

Overall, it is safe to say that Piezo2 ion channels dominantly guard homeostasis in somatosensory neurons, while Piezo1 ion channels maintain homeostasis in neuromodulator peripheral cells. Accordingly, the Piezo1 channels in peripheral tissues serve the purpose of cellular mechanoreceptors, and they could be neuromodulators through cross-talk with the sensory Piezo2 ion channels, which are the homeostatic gatekeepers of the central nervous system (CNS) [56].

### 3.2. Piezo2 Microinjury

Recently, it was hypothesized that acute stress response (ASR) induced, fatiguing eccentric contraction-based mechano-energetic microdamage of Piezo2 channels in proprioceptive sensory terminals could open a path to pathophysiology on an autologous or noncontact basis, as suggested, for example, in the mechanism of delayed onset muscle soreness (DOMS), noncontact anterior cruciate ligament (ACL) injury and post-orgasmic illness syndrome (POIS) [8,9,53]. Since the critical gateway to pathophysiology is proposed to be initiated at neural loci, neural interpretation and tracing of the pathophysiology from the neuronal angle could serve us with a better clinical understanding in the future. Notably, this type of somatosensory terminal impairment caused by damaging eccentric contractions is proposed to be analogous to the so-called TAD, often seen in platinum-analogue and paclitaxel chemotherapy [8,57]. These TAD lesions do not result in classical Wallerian-type neuronal degeneration [8,16,19]. Furthermore, it has been demonstrated that this neurotoxic effect of oxaliplatin impairs only the static phase firing sensory encoding and hardly affects the dynamic sensory component [19].

If harsher tissue injury with polymodal Aδ and C-fiber sensory contribution follows the pain-free primary proprioceptive Piezo2 microinjury, then the clinical picture of an acute compression sensory axonopathy could evolve with a delayed onset of pain sensation [8,9]. ASR-induced axon terminal mitochondrial mechano-energetic deficiency could also impair glutamate vesicular release, leading to glutamate spillover at the affected peripheral proprioceptive terminals [9,31]. Furthermore, the microdamaged Piezo2 channels are theorized to become “leaky” to Piezo currents and even to glutamate in a supposedly inactivated state [9]. Notably, the pores of Piezo channels are nonselective to cations; however, Piezo currents slightly favor Ca^2+^ [46,58]. In line with this theory, recent animal research on mice showed that glutamate vesicular release is essential for the maintenance of muscle spindle afferent excitability, especially during stretch maintenance, but not for dynamic sensitivity [27]. Since Piezo2 ion channels are also found to be the primary mechanotransduction channels for proprioception [28], the aforementioned impairment of muscle spindle-derived static phase firing sensory encoding could be expected due to the proposed Piezo2 microinjury [9,59]. Finally, glutamate excitotoxicity at the peripheral terminal could activate N-methyl-D-aspartate (NMDA) receptors at the other axonal endings of the same pseudounipolar proprioceptive neurons, namely, at the presynaptic central terminal on the spinal dorsal horn [31,60,61]. Notably, Murase et al. also suspected the involvement of activated NMDA receptors in the DOMS mechanism [23].

Finally, Alvarez et al. showed that in the case of peripheral nerve injury, the VGLUT1 synapses of Type Ia central terminals are lost permanently on motoneurons, leading to a loss of proprioceptive feedback from the muscle spindles [21]. Sonkodi et al. proposed a similar disruption, however acutely or transiently, in VGLUT1 transmission on motoneurons in DOMS when transient Piezo2 channelopathy is present [22]. As a result of the lost VGLUT1 synapses, the NMDA receptors at the axonal endings of the same pseudounipolar proprioceptive neurons could be activated, namely, at the presynaptic central terminal on the spinal dorsal horn [31,60,61]. Indeed, Ia monosynaptic excitatory postsynaptic potentials do not involve NMDA receptor activation substantially under homeostatic circumstances [62]. However, it is important to note that the inactivation of Piezo ion channels is a physiological response to hyperexcitation and is within homeostasis [26,63]. For example, other ion channels, such as noxious heat and capsaicin-activated TRPV1, have a role in Piezo2 channel inactivation [64]. Moreover, stress-induced modified kinetics of Piezo are also known in pathology [63]. Correspondingly, it has been suggested that Piezo2 could transiently transform from a physiological state to a pathophysiological state in hyperexcited proprioceptive terminals under an induced ASR when unaccustomed or strenuous eccentric contractions should be sustained, which could be the aforementioned “leakiness” to Piezo currents and even to glutamate when it should not be [9]. Correspondingly, Sonkodi et al. suggested that proprioceptive Piezo2 microdamage and the loss of the VGLUT1 connection could involve NMDA receptor activation [9,22].

Notably, various leakage currents could be sidetracked from the main logic pathway of currents, and subthreshold current is one major type of leakage current. The current author suggests that the leakiness of Piezo2 microinjuries could induce such imbalanced subthreshold currents due to defective conversion of steady depolarization into repetitive firing, as was proposed in platinum-analogue chemotherapy [65].

The current author is emphasizing the difference between two states, namely, that proprioceptive terminal hyperexcitation without leakiness leads to inactivation of Piezo2, as opposed to hyperexcitation with leakiness that could lead to pathology and transient Piezo2 channelopathy [9]. Furthermore, it is suggested that the repetitive reinjury or permanent microdamage of these Piezo2 channels could lead to increased sensitivity longitudinally due to primary TAD-like lesions [9,59]. Accordingly, platinum-analogue and paclitaxel chemotherapy-induced TAD lesions are observed in an acute and chronic way as well [16,19].

One explanation for the aforementioned transient Piezo2 microinjury theory [9] could be learned from the favorable effect of diclofenac on DOMS [7,66]. Notably, proprioceptive Piezo2 channelopathy is suspected to be the critical cause of DOMS [9]. Diclofenac is a dual-action nonsteroidal anti-inflammatory drug that inhibits both the cyclooxygenase and lipoxygenase pathways [7,66]. The positive effect of diclofenac could be translated as the attenuation of proprioceptive hyperexcitation through the inhibition of the cyclooxygenase pathway and stabilization of membrane lipids around the Piezo2 ion channel through the inhibition of the lipoxygenase pathway. Piezo2 ion channels are indeed bordered by lipids in the cell membrane [43] and deformed by lipid bilayers [45]. Importantly, a force-from-lipid hypothesis exists to explain the force-coupled gating of these types of mechanosensitive channels [46,67]. Accordingly, abrupt elevation of the tension of these lipid bilayers could open these mechanosensitive channels [67]. However, the hyperexcited Piezo2 channels could be inactivated, but the ASR-derived excessive lipid peroxidation could indirectly destabilize the structures of the labile hyperexcited and inactivated Piezo2 channels and, as a result, could become “leaky” to Piezo imbalanced subthreshold currents and possibly even to glutamate [9] when they should not be. Another explanation could be that the phospholipid substrate PIP2 of myotubularin-related protein-2 is excessively damaged by the ASR-derived heightened lipid peroxidation activity, since these phospholipids participate in the control of Piezo2-dependent mechanotransduction [68]. The current author proposes that this is why eicosapentaenoic acid-rich fish oil supplementation could have a favorable effect on DOMS prevention, as demonstrated by Ochi et al. [69]. Another significant underlying factor could be that the excitatory functioning of Piezo channels depletes membrane cholesterol locally [70,71]. In addition, dysfunctional mitochondria trafficking and mitochondrial mechano-energetic depletion are suspected, as in platinum-analogue and paclitaxel chemotherapy [16,19], at the proprioceptive terminals in the proposed ASR-derived TAD-like lesion of noncontact injuries, such as DOMS due to fatiguing forced lengthening contractions [8].

### 3.3. Impaired Proprioception, Static Phase Firing Encoding and Delayed MLR

The signs of the suggested silent proprioceptive terminal Piezo2 microinjury could be impaired proprioception and transient autonomic disbalance [9,31]. The grounds of proprioceptive impairment were suggested and lately demonstrated to be a minor alteration of the stretch reflex in the affected striated muscles [22,31,53]. This impairment is attributed to the aforementioned compromised static phase firing sensory encoding of the stretch reflex [22,31,32,53]. Accordingly, it has been observed that stretch-evoked static firing in muscle spindle primary mechanosensory terminals is more sensitive to glutamate than dynamic firing [26]. Furthermore, glutamate vesicular release is essential for muscle spindle primary afferent excitability in stretch maintenance but not for dynamic sensitivity, as was noted previously [27]. This Type Ia afferent terminal Piezo2 channelopathy could cause some of the monosynaptic static phase firing sensory encoding of the stretch reflex to be altered to the secondary Type II fiber-induced polysynaptic pathways, and this could be the basis of the proposed delayed latency of the medium latency response (MLR) of the stretch reflex and the reduced range of motion [22,31,32,53]. As a result, the constantly firing, non-adapting Type II static impulses could arrive earlier on the spinal dorsal horn than the microinjured adapting Type Ia sensory inputs [31]. This silent exchange of static phase firing sensory encoding could be the explanation for the compensatory exaggerated contractions and reduced range of motion [31], as in the case of paclitaxel-based chemotherapy [18].

Accordingly, there could be three consequences of the proposed transient Piezo2 microinjury and the VGLUT1 synaptic disconnection on motoneurons. First, Piezo2 channelopathy-induced imbalanced subthreshold currents and the resultant defective conversion of steady depolarization into repetitive firing could be the reason why DOMS delays the M-wave latency for 2 days after exercise, as reported by Kouzaki et al. [17]. Second, glutamate signaling is switched from vesicular-based to activated NMDA receptor-based signaling, which could be the equivalent of the aforementioned switch from monosynaptic Type Ia static firing to polysynaptic Type II static firing. Third, the lost VGLUT1 sensory encoding and the activated NMDA receptors could induce NaPICs or, even more likely, NMDA PICs [72] on motoneurons, leading to exaggerated contractions and reduced range of motion, as was suggested by Sonkodi [57], demonstrated by Del Rosario et al. [57] and could be observed after nerve crush injury [73,74].

In summary, the ASR-related microinjury of the Piezo2 channels at the proprioceptive terminal, which is analogous to the compromised primary preprogram of postural control, could lead to a switch to a secondary preprogram of postural control in the form of exaggerated contractions and reduced range of motion to enhance postural control and to provide supranormal protection against gravity and shock attenuation [9,31,53]. For the time being, proprioception is impaired, because the neuro-energetic demand of the compensatory preprogram of postural control is so high segmentally that it could lead to lost control of other unaffected segments due to the resource limitation of the overall proprioceptive system [31].

The mechano-energetic loading is suggested to accumulate in a dose-limiting manner, as is observed in TAD lesions [16], due to the autologous superposition of compression and shear forces [8,53]. This mechano-energetic overloading could eventually lead to the stress-derived terminal lesion of the Type Ia proprioceptive sensory neurons innervating the muscle spindles. Correspondingly, it could impair the static phase firing sensory encoding of eccentric contractions.

### 3.4. Transient Autonomic Disbalance

When fatigued muscle is not capable of sufficient force production but muscle performance should be maintained or heightened cognitively, then “overreaching” is induced to accomplish the desired exercise task. Coaches and athletes repeatedly use this “overreaching” process as a goal in training and learning sessions [31,53,75,76]. Moreover, repetitive “over-reaching” training sessions with adequate recovery periods could develop a higher level of homeostasis due to acute adaptation. This elevation from resting homeostasis is called “supercompensation” [31,53,77]. The “overreaching” response and “supercompensation” are guided by the autonomic nervous system [76], and this process entails the use of motor learning and memory in accordance with the proprioceptive system [31,53]. The aforementioned ASR is suggested to be a homeostatic driver in this “overreaching” response when force production is depleted in strenuous or unaccustomed eccentric exercise moments, but the performance should be maintained or heightened to accomplish the cognitive demand-derived exercise task [31,53]. Piezo2 channels at the proprioceptive sensory terminal are proposed to exhibit a critical path in the osteocalcin-induced “overreaching” ASR time window. In this state, the inactivation of Piezo2 channels is a physiological response to hyperexcitation and is still within homeostasis [8,9]. However, this “over-reaching” response could go overboard, leading to the previously mentioned hyperexcitation with leakiness, which is the proposed transient Piezo2 channelopathy.

Interestingly, complete cardiac parasympathetic activity measured by heart rate variability (HRV) can return to pre-exercise levels only within 1 to 2 days; accordingly, the number of days positively correlates with higher exercise intensities [76]. This transient autonomic imbalance time interval overlaps the proposed transient Piezo2 channelopathy in DOMS [9]. Correspondingly, this time overlap could have two implications. First, an intimate crosstalk between the autonomic nervous system and the Piezo2 ion channels could exist, as is insinuated by Abboud and Sonkodi et al. [9,78], and as a result, the transient dysfunctionality of the microinjured Piezo2 could induce a transient autonomic disbalance. Even more precisely, the cardiac parasympathetic activity after an ASR, which is a full parasympathetic withdrawal [30], cannot return until the Piezo2 channelopathy is functionally regenerated or becomes unleaky in the hyperexcited states. Correspondingly, the current author suggests that the mechano-energetically dysfunctional proprioceptive terminal mitochondria, impaired glutamate vesicular release, imbalanced subthreshold currents and the resultant NMDA receptor activation due to Piezo2 microdamage are responsible for the transient autonomic disbalance in DOMS. Indeed, animal research in rats showed that activation of NMDA receptors reduced HRV and induced cardiac autonomic imbalance [79]. Second, the aforementioned intimate cross-talk between Piezo1 and Piezo2 ion channels should also exist, more likely in the form of Piezo currents, and the microinjury of Piezo2 could alter the functionality of this communication.

Overall, it is safe to address a Piezo system instead of individual Piezo channels, which is even more than the proprioceptive system and based on the aforementioned implication of cross-talk between Piezo channels [56]. Notably, this Piezo system appears to principally contribute to the regulation of proprioception, postural orientation, orthostasis and the alignment of cells, tissues and organs. An indication of this notion is the finding that Piezo1 channels sense and respond not only in a spatially restricted manner [80] but also throughout the entire body holistically to enhance performance and reset cardiovascular homeostasis accordingly [81]. However, pain-free proprioceptive Piezo2 channelopathy, which also means the impairment of the static phase firing sensory encoding of the affected stretch reflex, opens the gateway to pathophysiology and could lead to impaired postural control and impaired orthostasis. In addition, humans seem to have a secondary preprogrammed compensatory pathway for these noncontact primary proprioceptive terminal microinjuries.

## 4. Noncontact Injury Mechanism of DOMS

Proprioception, described as our sixth sense by Sir Charles Bell in 1830 [82], is a mysterious, diagnostically challenging system referring to the sense of the positions and actions of the extremities and providing our postural control effortlessly [46]. It is often the no man’s land in clinical medicine because it rather pertains to the peripheral nervous system; however, it has profound preprogrammed pathways in the central nervous system. Morgan et al. [83] and Hody et al. [84] identified earlier the dichotomous injury mechanism of DOMS. The biphasic acute proprioceptive compression axonopathy or neuronal noncontact injury mechanism was first described through the new noncontact neuroinflammation theory of DOMS [8,9].

### 4.1. Primary Injury Phase or Piezo2 Channelopathy

The proprioceptive primary microinjury is hypothesized to occur when ASR-induced energy depletion at the primary afferent’s peripheral terminal prevails, and as a result, the mechano-energetically dysfunctional mitochondrial supply could impair the glutamate vesicular release system in addition to the autologous microinjury of the proprioceptive Piezo2 ion channels [8,9,31]. The realization that these types of lesions could behave in an analogous way that is experienced as a side effect of axonopathy-causing paclitaxel and platinum-analogue chemotherapy could enhance our understanding because it evolves in a dose-limiting, threshold-driven manner and is not associated with classical Wallerian axonal degeneration [8,9,16,19,31]; however, it could disrupt the monosynaptic static phase firing sensory encoding on motoneurons [19,20,21].

### 4.2. Secondary Injury Phase or Axonopathy

As a result of the primary noncontact microdamage, a secondary injury phase could succeed in the form of harsher tissue damage [8,53]. This more pervasive tissue damage in DOMS is due to the primary impairment of proprioceptive protection, as we could experience in other noncontact injuries [8,31,53,57,85]. The pivotal involvement of other sensory neurons, such as nociceptive neurons [24], and other ion channels, e.g., TRPV1 and TRPV4 [25], could occur in this stage of the noncontact pathophysiology of DOMS [8]. Notably, nociceptive C-fiber involvement in mechanical hyperalgesia of DOMS could be correspondingly secondary but pivotal because these neurons contribute to the slow temporal summation of pain [8,24,86] (see Table 1).

Recent research is evolving in support of the noncontact neuroinflammation theory of DOMS [8,9,42]. Muscle spindle-derived proprioceptive large fiber involvement has been demonstrated in DOMS, as suggested by the acute proprioceptive compression axonopathy theory [8,12]. Moreover, eccentric exercise-derived damage, implicated within the muscle spindle, reduces proprioception immediately after DOMS-inducing exercise and not with a delayed onset [2]. The current author interprets these findings to mean that muscle spindle-derived proprioceptive terminal Piezo2 microinjury is the critical gateway to pathophysiology in DOMS, but mechanical hyperalgesia cannot evolve without the secondary, harsher tissue injury and resultant C-fiber contribution [9] (see Table 1).

A secondary preprogrammed compensatory pathway could come into play as a consequence of the primary noncontact Piezo2 microdamage to enhance postural control, enhance shock attenuation and support the body against gravity at the injured segmental level [9,31,53]. The basis for the switch to this secondary compensatory pathway is the aforementioned silent exchange of static phase firing sensory encoding and the heightened inducement of NaPICs or, even more likely, NMDA PICs [72] on motoneurons, resulting in compensatory exaggerated contractions and reduced range of motion [31].

Notably, the lost function of Piezo channels indeed induces exaggerated mechanoreflexes and contractions in compromised Aδ or Type III sensory endings [87]. However, the impaired Type III fiber-associated compensatory exaggerated contractions and reduced range of motion induced from extracellular matrix or muscles are suggested to evolve only in the secondary injury phase of DOMS, as hypothesized by the acute autologous proprioceptive compression axonopathy theory [8,31].

Accordingly, the C-fiber pain pathway is interlinked with Type III fibers during the secondary phase of the DOMS mechanism [8] due to muscle or extracellular matrix damage. Indeed, chemical and enzymatic destruction of the extracellular matrix impairs Piezo2 mechanogating putatively [46,88], and the deep fascia seems to be more sensitive to noxious chemical stimuli than skeletal muscles in DOMS [89]. However, it cannot be the primary damage because the impairment of proprioception could be experienced immediately after DOMS-inducing exercise, and certainly, the secondary damage is not muscle spindle derived [2] (see Table 2). It is important to note that muscle spindles cannot be viewed as entirely isolated anatomical structures, but rather as a continuum with extrafusal space. Most intrafusal muscle fibers extend beyond the ends of the muscle spindle capsule and are clearly attached to the surrounding connective tissue [90]. Furthermore, it is speculative but likely that numerous transverse connections exist from the intracellular space across the cell membrane to the extracellular matrix and between extrafusal fibers, distal muscle spindle capsules and terminating intrafusal fibers [91]. The current author suggests that a good candidate for this extracellular matrix-based trans-spindle cross-talk between Type Ia Piezo2 and Type III Piezo2 channels could be the Piezo1 ion channels. Correspondingly, cellular traction forces produce spatially restricted Piezo1-mediated Ca^2+^ flickers on a noncontact or no external mechanical force basis [80]. Notably, the pores of Piezo channels are nonselective to cations; however, Piezo currents slightly favor Ca^2+^ [46,58], and this preference could have special importance in Piezo2-based sensing of these Ca^2+^ flickers, especially in a “leaky” microinjured state. Indeed, recent findings put forward a force-from-filament or tether model based on Piezo channels tethered to the actin cytoskeleton, and the perturbation of this tethering could impair Piezo-transduced feedback [92].

The current author interprets these findings to mean that enzymatically impaired Piezo2 mechanotransduction of Type III fibers exert an additional, but this time delayed onset presynaptic inhibitory, effect on the already mechano-energetically microinjured muscle spindle-derived proprioceptive central terminals. This mechanism could be analogous to that presented by Fernández-Trillo et al., where the eye blinks of Piezo2 knockout mice were lower when von Frey filaments were applied to corneal Aδ sensory fibers compared to those of wild-type mice [93]. Notably, the short latency blink reflex is induced by the stretching of extraocular muscles and elicited in the extraocular muscle spindles [94].

In summary, polymodal Aδ fibers that contain Piezo2 ion channels could be good candidates for cross-talk with nociceptive C-fibers in the secondary phase of the DOMS mechanism, as suggested earlier [8,31]. Notably, Borghi et al. found upregulated c-Fos at locations where proprioceptive primary afferents enter the spinal dorsal horn [42], further supporting the hypothesis and observation that the primary microdamage is rather at the Piezo2-containing Type Ia terminals [9,32], and the secondary damage is more related to Type III/C-fibers [8].

### 4.3. Tertiary or Longitudinal Injury Phase

Emerging evidence supports that DOMS has a tertiary or longitudinal injury phase, lasting up to a year, in the form of the repeated bout effect (RBE). RBE is a reduced DOMS effect of the initial one when the same exercise bout is repeated [9,31,53,95]. Accordingly, the initial bout of severe DOMS-inducing unaccustomed exercise comprising eccentric contractions could evoke reduced DOMS symptoms for at least 6 months, but this adaptation is lost within 9 to 12 months [96]. This “adaptation” phenomenon is attributed to neural, connective tissue and cellular mechanisms [5]. The current author proposes that the critical gateway to this “adaptation” process is the microinjury of the Piezo2 ion channels and is primarily orchestrated by the proposed intimate Piezo current-based cross-talk between Piezo2 and Piezo1 channels involving the sensory neurons, connective tissue, or more precisely, the extracellular matrix and peripheral cells. Notably, recent studies are emerging in support of this autologous injury mechanism theory since the aforementioned myosin-II-mediated cellular traction forces could induce spatially restricted Piezo1-transduced Ca^2+^ flickers even in the absence of external mechanical force [80]. Furthermore, the suggested proprioceptive Piezo2 channelopathy-induced NMDA receptor activation opens several memory pathways at the central terminal on the spinal dorsal horn, such as pain memory, inflammation, working and episodic memory [31]. This NMDA receptor activation process is in addition to the one that is suggested to be the result of ASR inducing osteocalcin in the CNS [31]. Notably, osteocalcin exerts a strong influence on spatial learning and memory [97].

## 5. Oxidative Stress and Redox Imbalance

The relevance of reactive oxygen species (ROS) production in DOMS has been established, although with a temporal disassociation [98]. Earlier, the source of production was attributed solely to inflammatory agents in the muscle [99]. Correspondingly, free radicals are involved in the degeneration process of removing the damaged muscle. Furthermore, free radicals also have a role in the regeneration process as signaling molecules to regulate muscle cell growth, remodeling, differentiation, and proliferation [100].

Importantly, the nervous system is highly susceptible to free radical damage due to its high energetic demand [101,102]. Nitric oxide has a protective nourishing effect on neurons through vasodilatation, yet its radical could harm their proteins, lipids, and cells [103], even leading to energetic failure [104] and apoptosis [105,106]. The accumulation of these radicals could enhance nociception and instigate distal degeneration of nerve fibers [16]. The noncontact acute proprioceptive compression axonopathy theory of DOMS implied already that ASR-derived autologous superposition of compression forces could possibly cause such a severe mechano-energetic insult on axonal mitochondrial trafficking to the proprioceptive terminals of the muscle spindles that microdamages the axon’s energy supply [57], which is akin to the aforementioned TAD-like lesion [16].

Nevertheless, the oxidative damage and redox imbalance affecting the spinal cord and Type Ia proprioceptive terminal could be evidence of neuroinflammation. Accordingly, recent findings demonstrated that intense acute swimming (IAS) induces oxidative stress in the spinal cord, and the resultant redox imbalance was shown to have a role in IAS-associated hyperalgesia and nuclear factor-kappa B (NF-kappa B) activation [107]. In line with this finding, Borghi et al. presented earlier in mice that unaccustomed IAS indeed increased the level of superoxide anion, lipid peroxidation and oxidative stress in the spinal cord at the height of DOMS [42]. Moreover, intrathecal administration of glial and NF-kappa B inhibitors could diminish superoxide anion production and lipid peroxidation, as well as overall oxidative stress and pain [42]. Notably, astrocytes and microglia can elevate ROS generation under pathological conditions [31,42,108,109]. The current author proposes that primary afferent peripheral terminal hyperexcitation with “leakiness” at Piezo2 is such a pathological condition in DOMS. Accordingly, it is not surprising that platinum-analogue drugs also elevate ROS production as the primary mechanism of platinum-induced peripheral neuropathy [110] with the activation of the NF-kappa B pathway [111].

ROS-induced NF-kappa B activation might also have an analogous role in the periphery in NGF upregulation in muscle cells [23]. Notably, NGF has a key role in DOMS maintenance because the time window of NGF upregulation correlates with the time window of DOMS with the same delay [23]. This upregulation of NGF is propelled by B_2_ bradykinin-induced NF-kappa B activation on muscle cells [23]. Interestingly, bradykinin is known to upregulate Piezo2 currents and essentially contributes to mechanical hyperalgesia [23,43,112]. Bradykinin could enhance the permeability of the muscle spindle’s selective membrane capsule under the proposed ASR-induced pathological hyperexcitation [8] and, therefore, possibly contribute to lipid peroxidation and eventually to the suggested Piezo2 microinjury [9]. Furthermore, NF-kappa B activation also has a role in oxidative metabolism through nicotinamide adenine dinucleotide phosphate (NADPH) [42,113].

In summary, unaccustomed or strenuous eccentric contraction-based pathological hyperexcitation of Type Ia afferents, proposed to be an acute Piezo2 channelopathy, induces ROS production-associated neuroinflammation and neuronal activation in the spinal cord of DOMS patients in addition to ROS generation in muscles.

## 6. Conclusions

Cognitive demand-derived acute stress response could microdamage the Piezo2 channels in an autologous manner of proprioceptive sensory terminals under repetitive unaccustomed or strenuous eccentric contractions. This over-reaching related mechano-energetic noncontact proprioceptive microdamage is proposed to be one principal gateway between physiology and pathophysiology in DOMS. The consequences of this proposed transient Piezo2 microinjury will be a VGLUT1/Ia synaptic disconnection on motoneurons, as we can learn from platinum-analogue chemotherapy. Furthermore, the Piezo2 channelopathy of these proprioceptive terminals seems to impair the static phase firing sensory encoding of the stretch reflex of the affected musculoskeletal system responsible for postural control. The secondary injury phase of DOMS with harsher tissue damage will be the result of primary proprioceptive impairment. The aforementioned fatiguing eccentric contraction-based pathological hyperexcitation of Type Ia afferents also induces ROS production-associated neuroinflammation and neuronal activation in the spinal cord of DOMS in addition to ROS generation in DOMS-affected muscles.

## Figures and Tables

**Table 1:** The two phases of DOMS adapted from the quad-phasic non-contact injury model [56].

Piezo2 Channelopathy Induced DOMS Injury Model
**Primary injury phase**
Repetitive superposition of compression forces due to fatiguing eccentric contractions
Fatigue and cognitive demand induced acute stress response
Acute stress derived energy depletion of the mitochondria in the affected proprioceptive terminals
Mechano-energetic impairment of Piezo2 and impairment of vesicular glutamate release
Painless Piezo2 channelopathy
**Secondary injury phase**
Harsher tissue damage due to impairment of Piezo2 with C-fiber contribution
Painful compression axonopathy

**Table 2:** Exercise-induced microdamages.

	Painless Condition	Exercise Induced Muscle Damage (EIMD)	Delayed Onset Muscle Soreness (DOMS)
**Primary Injury Phase in the Muscle Spindle**	Proprioceptive terminal microdamage	No proprioceptive terminal microdamage	Proprioceptive terminal microdamage
**Secondary Injury Phase**	No extrafusal microdamage and no C-fiber contribution	Extrafusal microdamage with C-fiber contribution	Extrafusal microdamage with C-fiber contribution
**Condition**	Painless microinjured state without DOMS lasting up to 2–3 days	Exercise-induced soreness without delayed onset	DOMS lasting up to 7 days

## Data Availability

Not applicable.
